# New fossil ephialtitids elucidating the origin and transformation of the propodeal-metasomal articulation in Apocrita (Hymenoptera)

**DOI:** 10.1186/s12862-015-0317-1

**Published:** 2015-03-13

**Authors:** Longfeng Li, Chungkun Shih, Alexandr P Rasnitsyn, Dong Ren

**Affiliations:** College of Life Sciences, Capital Normal University, 105 Xisanhuanbeilu, Haidian District, Beijing 100048 China; Paleontological Institute, Russian Academy of Sciences, 123, Profsoyuznayaul, Moscow, 117997 Russia; Department of Palaeontology, Natural History Museum, Cromwell Road, London, Sw7 5BD UK

**Keywords:** New genera, Jiulongshan formation, Daohugou, Yixian formation, Liutiaogou, Inner Mongolia

## Abstract

**Background:**

Apocrita has a special structure that its first abdominal segment has been incorporated into the thorax as the propodeum. The remaining abdomen, metasoma, is connected to this hybrid region via a narrow propodeal-metasomal articulation forming a “wasp waist”, which serves an important function of providing maneuverability, flexibility and posture for oviposition. However, the origin and transformation of the propodeal-metasomal articulation are still vague. Ephialtitidae, as the basal group of Apocrita from the Early Jurassic to the Early Cretaceous, have shown various types of propodeal-metasomal articulations.

**Results:**

This study describes and illustrates two new genera with three new species, *Acephialtitia colossa* gen. et sp. nov., *Proephialtitia acanthi* gen. et sp. nov. and *P. tenuata* sp. nov., collected respectively from the Early Cretaceous Yixian Formation at Liutiaogou and the Middle Jurassic Jiulongshan Formation at Daohugou, both in Inner Mongolia, China. These genera are assigned to the Ephialtitidae based on their complete wing venation, e.g. 2r-rs, 2r-m, 3r-m and 2 m-cu always present in the forewings and Rs, M and Cu in the hind wings. These new fossil ephialtitids have well-preserved propodeal-metasomal articulations indicating metasoma is broadly attached to propodeum.

**Conclusion:**

The broad articulation between the propodeum and metasoma in basal Ephialtitidae, likely passed on from a still more basal family Karatavitidae, suggests three separate pathways of the transformation of the “wasp waist” in three different derived lineages leading from Ephialtitidae to: (i) Kuafuidae and further to the remaining Apocrita, (ii) Stephanidae, and (iii) Evanioidea. In addition, the demise of ephialtitid wasps lagging behind the flourishing of angiosperms suggests that ephialtitid extinction might have been mainly driven by competition with numerous new taxa (eg. the abundant Cretaceous xylophilous Baissinae and Ichneumonoidea) appeared just before or/and soon after the J/K boundary.

**Electronic supplementary material:**

The online version of this article (doi:10.1186/s12862-015-0317-1) contains supplementary material, which is available to authorized users.

## Background

The extinct family Ephialtitidae was considered as the second most stem group in the suborder Vespina of Hymenoptera. Together with the most stem group of Karatavitidae, they jointly constituted the superfamily Ephialtitoidea [1]. In recent researches [2,3], Karatavitidae has been transferred to Orussoidea to form the stem group of Vespina, while Ephialtitidae moved to Stephanoidea as the stem group of Apocrita. The Stephanoidea was also treated as basal to Evanioidea and (Ceraphronomorpha + Proctotrupomorpha + (Ichneumonomorpha + Vespomorpha)). Ephialtitidae comprises two subfamilies: Ephialtitinae Handlirsh, 1906 and Symphytopterinae Rasnitsyn, 1980. Up to date, 29 genera with 77 species of ephialtitids have been described (Table 1). The lineage is known to exist from the Early Jurassic to the Early Cretaceous. It was nearly cosmopolitan in distribution, described from many countries, such as Kazakhstan, China, Spain, Germany, Russia, Mongolia, and Brazil, while about 70% of reported species from Kazakhstan [2-20]. Although ephialtitids are most likely parasitoids of xylophagous insect (beetle and/or horntail) larvae based on their long ovipositors and often trans-striate mesonotum, details of their biology are uncertain.

**Table 1:** **A list of Ephialtitidae fossils described from around the world**

**Genera (number of species)**	**Country (number of species)**	**Geological age (number of species)**	**References**
**Subfamily Ephialtitinae**			
*Acephialtitia* gen. nov. (1)	China (1)	K_1_ (1)	This paper
*Altephialtites* Rasnitsyn, 2008 (1)	Mongolia (1)	J_3_ (1)	Rasnitsyn (2008) [10]
*Asiephialtites* Rasnitsyn, 1975 (5)	China (1); Kazakhstan (4)	J_2_ (1); J_3_ (4)	Rasnitsyn (1975) [5];
			Rasnitsyn & Zhang (2010) [2]
*Cratephialtites* Rasnitsyn, 1999 (1)	Brazil (1)	K_1_ (1)	Rasnitsyn (1999) [9]
*Crephanogaster* Rasnitsyn, 1990 (2)	Russia (1); China (1)	K_1_ (2)	Rasnitsyn (1990) [8];
			Zhang et al. (2002) [19]
*Cretephialtites* Rasnitsyn, Ansorge, 2000 (1)	Spain (1)	K_1_ (1)	Rasnitsyn & Ansorge, (2000) [17]
*Ephialtites* Meunier, 1903 (1)	Spain (1)	J_3_ (1)	Meunier (1903) [4]
*Leptephialtites* Rasnitsyn, 1975 (10)	Kazakhstan (10)	J_3_ (10)	Rasnitsyn (1975) [5]
*Liadobracona* Zessin, 1981 (1)	Germany (1)	J_1_ (1)	Zessin (1981, 1985) [12,13]
*Mesephialtites* Rasnitsyn, 1975 (1)	Kazakhstan (1)	J_3_ (1)	Rasnitsyn (1975) [5]
*Montsecephialtites* Rasnitsyn, Delclòs, 2000 (1)	Spain (1)	K_1_ (1)	Rasnitsyn & Martínez-Delclòs (2000) [18]
*Parephialtites* Rasnitsyn, 1975 (1)	Kazakhstan (1)	J_3_ (1)	Rasnitsyn (1975) [5]
*Praeproapocritus* Rasnitsyn, Zhang, 2010 (2)	China (2)	J_2_ (2)	Rasnitsyn & Zhang (2010) [2];
			Li et al. (2013) [3]
*Proapocritus* Rasnitsyn, 1975 (6)	China (5); Kazakhstan (1)	J_2_ (5); J_3_ (1)	Rasnitsyn (1975) [5];
			Rasnitsyn & Zhang (2010) [2];
			Li et al. (2013) [3]
*Proephialtitia* gen. nov. (2)	China (2)	J_2_ (2)	This paper
*Sessiliventer* Rasnitsyn, 1975 (5)	Kazakhstan (5)	J_3_ (5)	Rasnitsyn (1975) [5]
?*Sinocephus* Hong, 1983 (? = *Proapocritus*) (1)	China (1)	J_2_ (1)	Hong (1983) [15]
*Thilopterus* Rasnitsyn et al., 2003 (1)	Germany (1)	J_1_ (1)	Rasnitsyn et al. (2003) [11]
*Tuphephialtites* Zhang et al., 2002 (1)	China (1)	J_2_ (1)	Zhang et al. (2002) [19]
*Sinephialtites* Zhang, 1986 (1)	China (1)	J_2_ (1)	Zhang( 1986) [14]
*Stephanogaster* Rasnitsyn, 1975 (7)	Kazakhstan (5); China (2)	J (5); J_2_ (2)	Rasnitsyn (1975) [5];
			Rasnitsyn & Zhang (2010) [2];
			Ding et al. (2013) [20]
**Subfamily Symphytopterinae**			
*Brigittepterus* Rasnitsyn et al., 2003 (1)	Germany (1)	J_1_ (1)	Rasnitsyn et al. (2003) [11]
*?Cephenopsis* Hong, 1984 (1)	China (1)	J_2_ (1)	Hong (1984) [16]
*Karataus* Rasnitsyn, 1977 (2)	Spain (1); Kazakhstan (1)	K_1_ (1); J (1)	Rasnitsyn (1977, 1978) [6,7];
			Rasnitsyn & Martínez-Delclòs (2000) [18]
*Karataviola* Rasnitsyn, 1975 (2)	Kazakhstan (2)	J_3_ (2)	Rasnitsyn (1975) [5]
*Micrephialtites* Rasnitsyn, 1975 (1)	Kazakhstan (1)	J_3_ (1)	Rasnitsyn (1975) [5]
*Symphyogaster* Rasnitsyn, 1975 (1)	Kazakhstan (1)	J_3_ (1)	Rasnitsyn (1975) [5]
*Symphytopterus* Rasnitsyn, 1975 (16)	Germany (1); Kazakhstan (15)	J_1_ (1); J_3_ (15)	Rasnitsyn (1975) [5]; Rasnitsyn et al. (2003) [11]
*Trigonalopterus* Rasnitsyn, 1975 (1)	Kazakhstan (1)	J_3_ (1)	Rasnitsyn (1975) [5]

Notes. J_1_. (Early Jurassic); J_2_. (Middle Jurassic); J_3_. (Late Jurassic); K_1_. (Early Cretaceous).

In this paper, we describe two new genera with three species, *Acephialtitia colossa* gen. et sp. nov., *Proephialtitia acantha* gen. et sp. nov. and *Proephialtitia tenuata* sp. nov., based on three well-preserved, nearly complete female specimens. These specimens were collected respectively from the Early Cretaceous Yixian Formation at Liutiaogou and the late Middle Jurassic Jiulongshan Formation at Daohugou, both are in Inner Mongolia, China. According to the accurate Ar–Ar and SHRIMP U–Pb dating, the age of the Yixian Formation is considered as the Early Cretaceous (late Barremian, about 125 Ma) [21,22]. The age of the Daohugou fossil-bearing beds in the Jiulongshan Formation is the late Middle Jurassic (Bathonian–Callovian boundary, 165 Ma) [23].

## Methods

### Examined taxa and terminology

The type fossil specimens studied are housed in the Key Lab of Insect Evolution and Environmental Changes, the College of Life Sciences, Capital Normal University in Beijing, China. The specimens were examined and photographed, either dry or wetted with 95% ethanol, under Leica MZ 16.5 dissecting microscope (Leica, Wetzlar, Germany) with attached digital camera Leica DFC500. The specimens illustrated with the aid of camera lucida attached to the microscope. The figures are drawn by CorelDraw 12.0 and Adobe Photoshop CS5. Wing venation terminology is basically adapted from Rasnitsyn & Zhang [2].

### Phylogenetic analysis

In this study, we used the Xiphydriidae as an outgroup and thirteen taxa of suborder Vespina as ingroups to carry out the phylogenetic analysis. Twenty five characters were identified and scored for all taxa. The matrix used is that of Rasnitsyn & Zhang [2] with addition of the new characters of Ephialtitidae. A complete list of the taxa (Additional file 1: Table S1) and the character state matrix (Additional file 2: Table S2) used in the phylogenetic analysis are provided. The phylogenetic analysis was carried out in *NONA* [24] in conjunction with *WinClada* [25]. Tree searches were performed using an heuristic search method (options: set to hold 10 000 trees, 1000 replications, 100 starting tree replication, multiple TBR + TBR search strategy). Character codings were set up by using Nexus Data Editor 0.5.0 [26] with all characters unordered and of equal weight.

## Results

### Systematic paleontology

Order Hymenoptera Linnaeus 1758

Suborder Apocrita Gerstaecker 1867

Family Ephialtitidae Handlirsch 1906

*Acephialtitia* Li, Shih, Rasnitsyn & Ren, gen. nov.

urn:lsid:zoobank.org:act:8B0E02F4-A59C-494B-B91F-7960E665594F

#### Etymology

The generic name is a combination of the Greek prefix ‘*ac*-’ (meaning needle and thorn) and the modified generic name ‘*Ephialtites*’. The gender is feminine.

#### Diagnosis

Body very large with long ovipositor for females. Antenna consisting of over 25 antennomeres, nearly as long as head and mesosoma combined. Wing venation nearly complete: forewing with 1-Rs reclival, shorter than 1-M, 1r-rs, 2r-rs, 2r-m, 3r-m, 2 m-cu present, 1r-rs very long, subparallel to RS + M, 2r-m and 3r-m subvertical, distant for much more than their length, cu-a slightly postfurcal, 2r-rs issuing from the mid-length of pterostigma, cell 1mcu in contact with 2rm by a point, cell 2rm shorter than 3rm, both much shorter than 1mcu, 2rm base distal comparing pterostigmal base; hind wing with Rs, M, Cu and r-m, 1-M gently curved, cu-a slightly postfurcal. Mesosoma long, not specialized. Metasoma broadly attaching to propodeum, metasoma slightly, smoothly widening rearwards. Legs slender, ordinary, trochantellus present. Ovipositor much longer than body, gently bent downwards.

#### Remarks

For comparison to other genera, see key below.

*Acephialtitia colossa* Li, Shih, Rasnitsyn & Ren, sp. nov.

urn:lsid:zoobank.org:act:BB8B0AB6-378C-4AB1-9B45-865B7FBF0D68

#### Diagnosis

As for genus by monotypy.

#### Etymology

The specific name is derived from the Latin word “*colossa*”, meaning very large, referring to its long body.

#### Holotype

**Figure 1:**
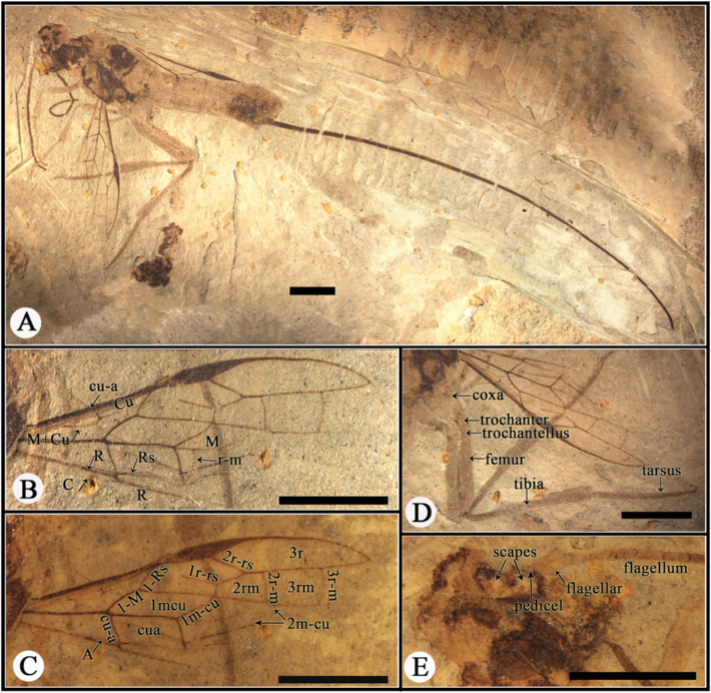
**Photographs of** ***Acephialtitia colossa*** **gen. et sp. nov.** Holotype, CNU-HYM-LB-2013004. **A**, Body with wings. **B**, Forewing. **C**, Interpretations of forewing with alcohol. **D**, Interpretations of hind legs. **E**, Interpretations of head with alcohol. Scale bars: 5 mm.

**Figure 2:**
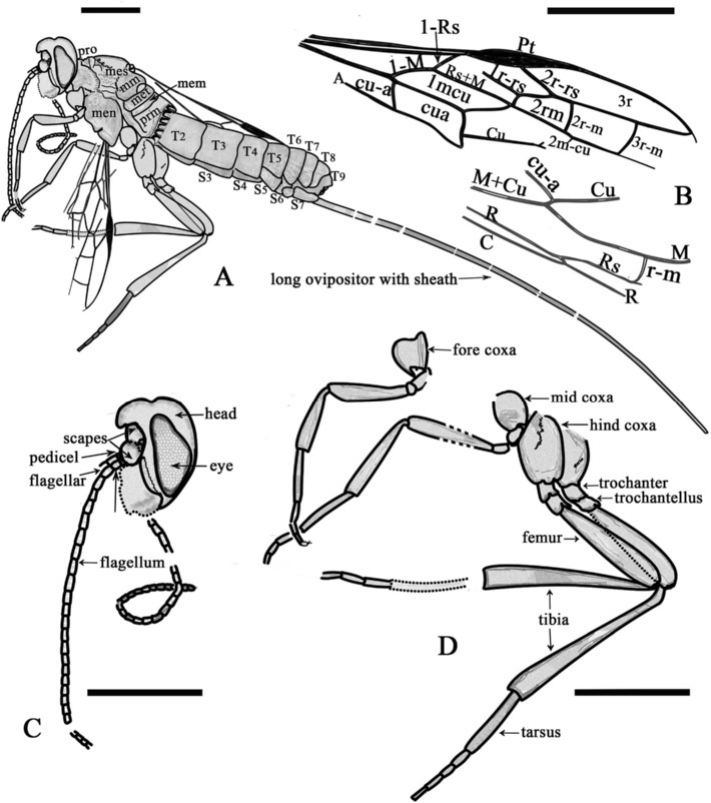
**Line drawings of** ***Acephialtitia colossa*** **gen. et sp. nov.** Holotype CNU-HYM-LB-2013004. **A**, Interpretations of body. **B**, Interpretations of wings. **C**, Interpretations of head. **D**, Interpretations of legs. Scale bars: 5 mm. pro, pronotum; prm, propodeum; mes, mesonotum; men, mesopleuron; met, metanotum; mem, metapostnotum; mm, mesoscutellum; T, Tergite; S, Sternite; Vf, Valvifer.

CNU-HYM-LB-2013004, female wasp, almost complete, preserved in lateral view, body and wings well-preserved and veins clearly discernible (Figures 1 and 2).

#### Horizon and locality

Early Cretaceous, Yixian Formation; Liutiaogou Village, Dashuangmiao Township, Ningcheng County, Chifeng City, Inner Mongolia, China.

#### Description

Body very long, about 28.3 mm long excluding ovipositor. Head and mesosoma mostly dark, antenna and metasomal apex darkened, legs and metasoma except apex pale.

Head short with a large eye in lateral aspect, 3.9 mm long and 5.7 mm high, about 1.5 times as high as long; antennal sockets ball-shaped, antenna consisting of over 25 segments, nearly as long as head and mesosoma combined; scape and pedicel poorly preserved, basal flagellomeres 2.5-3 times as long as wide, becoming slightly shorter and narrower apically.

Mesosoma approximately 9.4 mm long and 6.6 mm high, about 1.4 times as long as high; pronotum moderately short, mesonotum transversely ridged, medial suture and notauli not visible, transverse suture straight, axillae evident, mesoscutellum ca. 0.7 as long as mesoscutum; metanotum ca. 07 times as long as mesoscutellum, metapostnotum narrow but distinct, mesopleuron 4.8 mm long and 3.2 mm high, about 1.5 times as long as high; propodeum longer than metanotum and metapostnotum combined.

Legs nearly complete, fore leg nearly as long and wide as mid leg, but both shorter than hind legs; fore coxa and mid coxa rounded and small, hind coxa elongate-ellipse and big, about two times as big as fore or mid coxa; trochanter and trochantellus present in the fore leg, mid leg and hind legs; femora slender, fore and mid ones particularly so, hind femur about 4.7 times as long as wide; fore tibia shorter than fore femur, fore tarsus consisting of 5 segments much narrower than fore tibia, first segment distinctly longest; mid tibia nearly as long and wide as mid femur, tarsus with 4 segments preserved; hind tibia thin basally and gradually swollen toward apex, hind tibia longer than hind femur, about 1.5 times as long as hind femur, hind tarsus consisting of 5 segments, much narrower than hind tibia, first segment distinctly the longest.

Wings well preserved, forewing 14.9 mm long and 4.9 mm wide, with 1-Rs (1.1 mm) shorter than 1-M (1.8 mm) and longer than its distance (0.9 mm) to pterostigma; pterostigma long and narrow, about 6 times as long as wide; 2r-rs issuing from pterostigma slightly basad of its mid-length; 1r-rs long, directed to RS base (not to perostigma base), 2r-rs (1.7 mm) longer than maximal width of 2rm (1.2 mm); cell 2rm (2.4 mm) shorter than 3rm (2.7 mm); crossvein 2r-m (1.2 mm) shorter than 3r-m (1.5 mm), both crossveins slightly curved, subvertical; cell 1mcu in contact with 2rm by a point, 1mcu nearly parallelogram, 3 times as long as wide; 2 m-cu damaged, entering 3rm cell near its base; cu-a slightly postfurcal, nearly as long as 1-Rs, cell cua shorter than 1mcu but nearly as wide as that; hind wing with Rs, M, Cu and r-m preserved, 1-M long, gently curved, cu-a slightly postfurcal.

Metasoma broadly attaching to propodeum, with 8 segments visible, about 15.1 mm, segments 1–3 cylindrical, further rearwards becoming slightly, gradually wider, segments 1–2 of almost equal length, about 1.3 times as long as wide, remainder decreasing gradually in. Ovipositor slim and bent slightly downward with a sheath, approximately 50.6 mm long (1.8 times as long as body length of 28.3 mm).

#### Dimensions of holotype (in mm)

Body length 28.3 (female); length of head 3.9, high 5.7; length of antenna 13.5; length of mesosoma 9.4, width 6.6; length of pronotum 2.5, width 2.3; length of mesonotum 5.7, width 3.3; length of metanotum 1.0, width 3.2; length of metapostnotum 0.6, width 3.2; length of propodeum 2.4, width 4.2; length of metasoma 15.1, length of first metasomal segment 4.8, width 3.8, length of second metasomal segment 3.6, width 3.8; length of third metasomal segment 2.6, width 3.8; length of remaining metasomal segments 7.4, maximal width 4.8, length of valvifer: first 1.2, second 2.5, third 1.3; length of ovipositor 50.6; length of fore leg: coxa 1.5, trochanter 0.9, trochantellus 0.5, femur 3.8, tibia 3.2, tarsomeres I-V: 2.8, ?, 1.2, 0.4, 0.6; length of mid leg: coxa 2.0, trochanter 1.0, trochantellus 0.8, femur 4.4, tibia 4.8, tarsomeres I-V: 2.9, ?, 0.7, 0.6, 0.6; length of hind leg: coxa 3.8, trochanter 0.7, trochantellus 0.9, femur 6.2, tibia 9.0, tarsomeres I-V: 3.8, 1.0, 0.8, 1.0, 0.9.

*Proephialtitia* Li, Shih, Rasnitsyn & Ren, gen. nov.

urn:lsid:zoobank.org:act:8C5DB8DD-85FD-4DBC-9527-36F7268AD371

#### Etymology

The generic name is a combination of the Greek prefix ‘*pro*-’ (meaning the former) and a modification of the genus name *Ephialtit*es. The gender is feminine.

#### Diagnosis

Body very large with long ovipositor in female. Wing venation nearly complete: forewing with 1-Rs slightly reclival, shorter than 1-M and distinctly angular to it (for about 120°), and distant from pterostigma for almost 3 times its own length, 1r-rs short or absent; 2r-rs, 2r-m, 3r-m, 2 m-cu, cu-a and a_1_-a_2_ present, base of cell 2rm proximal in respect of pterostigma base, 2r-m and 3r-m subvertical, distant for much more than their own lengths, cu-a interstitial; hind wing with Rs, M, Cu and r-m, cu-a present, 1-M gently curved, cu-a slightly antefurcal. Ovipositor almost straight, much longer than body.

#### Remarks

For comparison to other genera, see key below.

*Proephialtitia acantha* Li, Shih, Rasnitsyn & Ren, sp. nov.

urn:lsid:zoobank.org:act:5C829AB1-EBC6-4028-95A1-E8AA5EBC69F2

#### Etymology

The specific name is derived from the Latin word “*acantha*”, meaning thorn or stinger, referring to its long and straight ovipositor.

#### Diagnosis

Forewing with 1r-rs absent, cell 2rm nearly as long as 3rm. First metasomal segment distinctly widening rearwards. Forewing with apex distinctly darkened in fore half only.

#### Holotype

**Figure 3:**
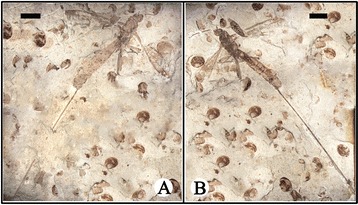
**Photographs of** ***Proephialtitia acantha*** **gen. et sp. nov.** Holotype CNU-HYM-NN-2014004. **A**, Part. **B**, Counterpart. Scale bars: 5 mm.

**Figure 4:**
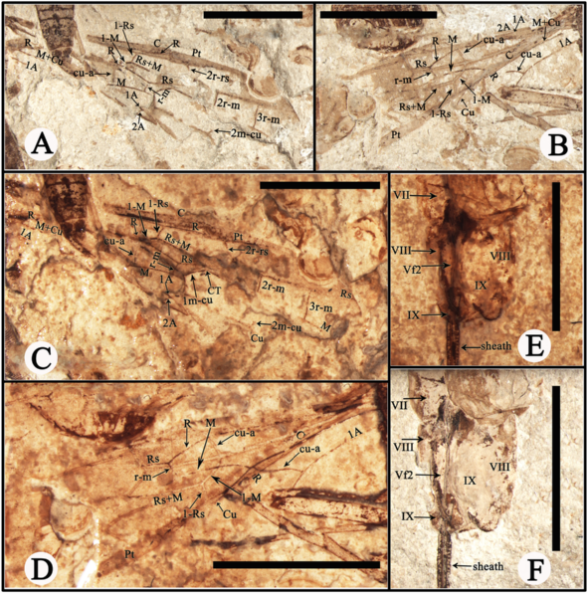
**Photographs of** ***Proephialtitia acantha*** **gen. et sp. nov.** Holotype CNU-HYM-NN-2014004. **A**, Interpretations of right forewing of counterpart. **B**, Interpretations of left forewing of counterpart. **C**, Interpretations of right forewing of counterpart with alcohol. **D**, Interpretations of left forewing of counterpart with alcohol. **E**, Interpretations of part of the matasoma with alcohol. **F**, Interpretations of part of the matasoma. Scale bars: 5 mm. VII, the seventh metasomal segment; VIII, the eighth metasomal segment; Vf2, the second Valvifer; IX, the ninth metasomal segment; OS, ovipositor sheath; OV, ovipositor.

**Figure 5:**
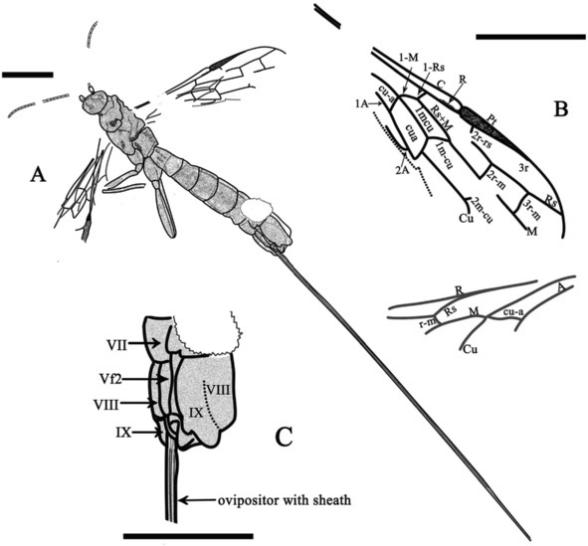
**Line drawings of** ***Proephialtitia acantha*** **gen. et sp. nov.** Holotype CNU-HYM-NN-2014004. **A**, Body with wings. **B**, Interpretations of wings. **C**, Interpretations of part of the matasoma. Scale bar: 5 mm. VII, the seventh metasomal segment; VIII, the eighth metasomal segment; Vf2, the second Valvifer; IX, the ninth metasomal segment.

CNU-HYM-NN-2014004, part and counterpart, almost complete female wasp preserved in slightly ventrolateral and dorsolateral aspect, body well-preserved but wings damaged (Figures 3, 4 and 5).

#### Horizon and locality

Late Middle Jurassic, Jiulongshan Formation; Daohugou Village, Inner Mongolia, China.

#### Description

Body very long, about 23.7 mm in length excluding ovipositor; head, antenna, mesosoma, 1st metasomal segment, hind femur and apex of mid femur dark, forewing apex infuscated in anterior part and hind wing apex infuscated.

Head medium sized, 1.8 mm long and 2.5 mm wide, about 1.4 times as wide as long; antenna slim with scape swollen, and pedicel distinctly narrower than scape, flagellum very thin.

Mesosoma approximately 6.4 mm long and 3.6 mm wide, about 1.8 times as long as wide; pronotum short, nearly as wide as head; mesonotum slightly wider than pronotum; propodeum long and broad, nearly as long as wide and in contact with mid and hind coxae.

Legs partly preserved, thin, trochanter and trochantellus present in the mid and hind legs, hind coxa much bigger than mid coxa; both mid and hind femora wider than their tibiae.

Wings preserved well, forewing 15.2 mm long and 4.0 mm wide as preserved, with first abscissa of Rs (1-Rs) 0.56 mm long, shorter than that of M (1-M), 1.0 mm; 1-Rs vertical to Rs and forming an angle of about 120° with 1-M at Rs + M, 1r-rs absent; cell 1mcu nearly paralleiogram, 2.6 times as long as wide; 2r-m and 2 m-cu partially preserved, 3r-m complete and slightly curved; cu-a interstitial, 0.9 mm long and slightly shorter than 1-M; cell cua nearly as long as 1mcu but slightly wider than that, cell 2rm nearly as long as 3rm but narrower than that; 2A incompletely preserved, meeting 1A before level of 1 m-cu; hind wing with Rs with r cell unknown to be closed or open, r-m distant from both RS and M bases, 1-M gently curved, cu-a (1.5 mm) S-like bent, slightly antefurcal and about 2.5 times as long as r-m (0.6 mm).

Metasoma about 15.5 mm long, broadly attaching to propodeum, with 8 segments visible, with nearly the same width from third segment to terminal; the first metasomal segment nearly trapezoidal in lateral view, about 1.6 times as long as maximal width, the second segment slightly wider but shorter than first; the third and fourth segments cylinder-shaped, the third segment nearly as wide and long as the fourth; remaining metasomal segments nearly as wide as the fourth segment. Ovipositor slim with a sheath, approximately 38.3 mm long as preserved (1.6 times as long as body).

#### Dimensions of holotype (in mm)

Body length 23.7 (female); length of head 1.8, width 2.5; length of mesosoma 6.4, width 3.6; length of propodeum 2.1, width 2.1; length of metasoma 15.5, length of first metasomal segment 3.2, maximal width 2.0, minimal width 1.1; length of valvifer 2.0, width 0.4; length of ovipositor 38.3.

*Proephialtitia tenuata* Li, Shih, Rasnitsyn & Ren, sp. nov.

urn:lsid:zoobank.org:act:C478CD96-B800-4383-BA45-EC517BCFEAAC

#### Etymology

The specific name is derived from the Latin word “*tenuata*”, referring to its long and straight ovipositor, gradually tapering from the base to distal end.

#### Diagnosis

Forewing with 1r-rs present as a short stub, cell 2rm much longer than 3rm. The first metasomal segment only slightly widening rearwards. Forewing with apex darkened throughout distal of 2r-m and 2 m-cu.

#### Holotype

**Figure 6:**
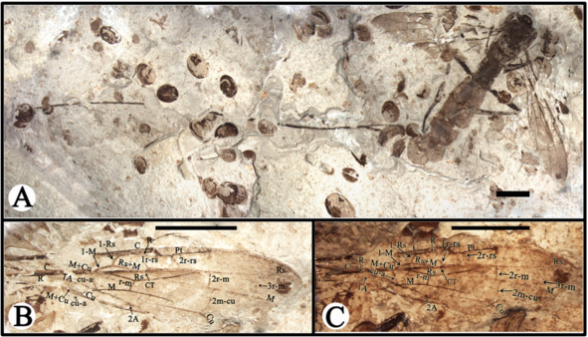
**Photographs of** ***Proephialtitia tenuata*** **sp. nov.** Holotype CNU-HYM-NN-2014005. **A**, Body with wings. **B**, Interpretations of wings. **C**, Interpretations of wings with alcohol. Scale bars: 5 mm. CT, cell 1mcu contact with 2rm at a point.

**Figure 7:**
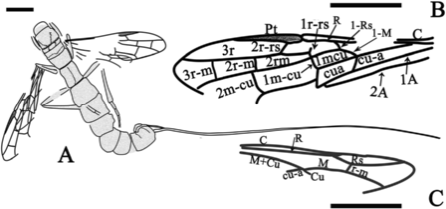
**Line drawings of** ***Proephialtitia tenuata*** **sp. nov.** Holotype CNU-HYM-NN-2014005. **A**, Body with wings. **B**, Interpretations of forewing. **C**, Interpretations of hind wing. Scale bars: 5 mm.

CNU-HYM-NN-2014005, almost complete female wasp preserved in dorsal view, body and wings nearly completely preserved and veins clearly discernible with alcohol (Figures 6 and 7).

#### Horizon and locality

Late Middle Jurassic, Jiulongshan Formation; Daohugou Village, Inner Mongolia, China.

#### Description

Body very long, about 32.1 mm long excluding ovipositor; color pattern similar to that of *P. acantha* gen. et sp. nov. except that legs and ovipositor darker and forewing apex distal of 2r-m ad 2 m-cu crossveins darkened throughout.

Head poorly-preserved

Mesosoma approximately 6.8 mm long and 4.3 mm wide, about 1.6 times as long as wide; mesonotum broad with median suture and notauli very wide, trans-costate, notauli widely diverging, contacting each other on transscutal suture, scutellum almost as long as mesoscutum; metanotum about half as long as scutellum; metapostnotum much shorter; propodeum broad and about as long as scutellum.

Legs partially preserved, hind leg very thin, hind femur slightly wider than hind tibia but shorter than that; hind tibia thin basally and gradually swollen toward apex, hind tarsus preserved with 3 segments, much narrower than hind tibia, first segment distinctly longer than any other segments of hind tarsus.

Wings well-preserved, forewing 20.2 mm long and 4.6 mm wide (as preserved), with 1-Rs (0.8 mm) shorter than 1-M (1.2 mm) and1-Rs forming an angle of about 120° with 1-M at Rs + M; 2r-rs issuing from pterostigma slightly basad of its mid-length; 1r-rs present but short (0.4 mm), nearly parallel to 2r-rs, 2r-rs (0.9 mm) longer than maximal width of 2rm (0.8 mm); cell 2rm (3.6 mm) distinctly longer than 3rm (2.8 mm); crossvein 2r-m (0.8 mm) shorter than 3r-m (1.3 mm), and 2r-m vertical, 3r-m slightly curved; cell 1mcu in contact with 2rm by a point; 2 m-cu complete, 1.6 mm long and 1.8 times as long as 1 m-cu (0.9 mm); cu-a interstitial, 1.0 mm long and slightly shorter than 1-M (1.2 mm), but slightly longer than 1-Rs (0.8 mm); 2A incompletely preserved, meeting 1A about at level of 1 m-cu; hind wing incompletely preserved, with r cell closed, r-m distant from both RS and M bases, 1-M gently curved, cu-a slightly antefurcal.

Metasoma broadly attaching to propodeum, with 7 segments visible, metasoma very long, about 23.6 mm; the first metasomal segment rectangle-shaped in dorsal view, about 1.2 times as long as wide, the second and third segment wider than the first. Ovipositor slim, approximately 52.7 mm long (1.7 times as long as body).

#### Dimensions of holotype (in mm)

Body length 32.1 (female); length of mesosoma 6.8, width 4.3; length of metasoma 23.6; length of first metasomal segment 4.0, width 3.3, length of second metasomal segment 4.3; length of third metasomal segment 2.9; length of ovipositor 52.7; length of hind leg: femur 5.7, tibia 7.1, tarsomeres I-III: 2.7, 1.1, 0.8.

#### Comparison

*P. tenuata* sp. nov. differs from the type species *P. acantha* sp. nov. in having forewing with 1r-rs present (vs. 1r-rs absent), cell 2rm much longer than 3rm (vs. nearly as long as 3rm), forewing apex infuscated completely (vs. only in anterior half), and first metasomal segment of nearly equal width throughout (vs. distinctly widening toward apex).

#### Key to genera of Ephialtitidae

(Unless stated otherwise, characters pertain to forewing)

Crossvein cu-a interstitial or near so (distant from M + Cu fork at most for 0.25 its length.
  Ovipositor long (sheaths much longer than valviver 2). Subfamily Ephialtitinae…………..2
  - Crossvein cu-a distinctly postfurcal (distant from M + Cu fork at least for 0.3 its length.
  Ovipositor short (sheaths not longer than valviver 2). Subfamily Symphytopterinae……..21
1-RS proclivous or subvertical……………………………………………………….……..3
  - 1-RS distinctly reclivous……………………………………………………………………9
1-RS distinctly proclivous………………………………………………………………………..4
  - 1-RS distinctly subvertical……………………………………………………..……………6
2-RS smoothly bent, with no trace of 1r-rs *…………………………………………….…………..*
  *………………………………………………Thylopterus* Rasnitsyn, Ansorge & Zessin, 2003 [11].
  - 2-RS angular at junction with 1r-rs…………………………………………………..……..5
2A complete with rudiment of basal loop. 1r-rs complete…………………………………..
  ………………………………………………*Praeproapocritus* Rasnitsyn & Zhang, 2010 [2].
  - 2A straight or incomplete basally. 1r-rs incomplete or rudimentary…………………………
  …….*Proapocritus* Rasnitsyn, 1975 [5] (*Cephenopsis* Hong, 1983 possibly runs here as well)
(4). 3r-m and 2 m-cu lost, 1a-2a present………………… *Parephialtites* Rasnitsyn, 1975 [5]
  - 3r-m and 2 m-cu present, 1a-2a lost…………………………………………………………7
Cells 2-3rm short, 3rm shorter than 1mcu……………… *Crephanogaster* Rasnitsyn, 1990 [8]
  - Cell 3rm much longer than 1mcu……………………………………………………..……..8
Forewing 5 mm or longer. Hind wing when known with r cell closed and 1-M longer and
  distinctly bent…………………………………..……….*Stephanogaster* Rasnitsyn, 1975 [5]
  - Forewing 4 mm or shorter. Hind wing when known with r cell closed and 1-M shorter and
  straight (or nearly so)…………………….……..…………*Asiephialtites* Rasnitsyn, 1975 [5]
(2). 3r-m and 1a-2a lost…………………………………………………………………….10
  - 3r-m present………………………………………………………………………………11
2 m-cu lost, 2-RS angular at junction with rudimentary 1r-rs………………………………
  *…………………………….…………………………………………Liadobracona* Zessin, 1981 [12]
  - 2 m-cu present, 2-RS almost straight with no sign of 1r-rs ……*Sessiliventer Rasnitsyn*, 1975 [15]
(9). 2r-m lost, 1a-2a lost…….. *Montsecephialtites* Rasnitsyn & Martínez-Delclòs, 2000 [18]
  - 2r-m present ……………………………………………………………………………….12
2r-m oblique, sinuate, distant from 3r-m for about its own length or less………………..13
  - 2r-m subvertical, not sinuate, often distant from 3r-m for much more than its length…….15
2r-rs and 2r-m practically coincide, 1a-2a present…..*Tuphephialtites* Zhang et al. 2002 [19]
  - 2r-rs and 2r-m clearly distant, 1a-2a lost …………………………………….……………14
Cell 2rm receiving 2cu-a…………………………..……*Cratephialtites* Rasnitsyn, 1999 [9]
  - Cell 3rm receiving 2cu-a ……………………*Cretephialtites* Rasnitsyn & Ansorge, 2000 [17]
(12). Cell 2rm long, surpassing level of pterostigmal base basally. Hind wing with 1-RS vertical to both R and 2-RS. Ovipositor evenly bent upward…......*Ephialtites* Meunier, 1903 [4]
  - If (rarely) cell 2rm as above, hind wing with 1-RS oblique. Ovipositor never bent upward…...16
1r-rs complete, reaching base of pterostigma……………….*Sinephialtites* Zhang, 1986 [14]
  - 1r-rs not reaching base of pterostigma ……………………………………………………17
1r-rs long, almost parallel to RS + M………………………………. *Acephialtitia* gen. nov.
  - 1r-rs short or lost ………………………………………………………………………….18
Cell 3rm distinctly shorter than 1mcu, 2rm still shorter, 2-RS straight, with no sign of 1r-rs,
  hind wing with 1-M straight, 1a-2a present………………*Altephialtites* Rasnitsyn, 2008 [10]
  - Cell 3rm not shorter than 1mcu, 2-RS bent or angular, often with 1r-rs, hind wing with 1-M
  bent…………………………………………………………………………………………19
1-RS short, straight, strictly angular with 1-M, distant from pterostigma for more than twice its length, metasoma long, narrow, with 1st segment much longer than wide………………..
  *……………………………………………………………………………..……Proephialtitia* gen. nov.
  - 1-RS more or less aligned with 1-M, more or less bent, less distant from pterostigma,
  metasoma less elongate……………………………………………………………………….20
Antenna 12-segmented, 1a-2a present, metasoma widest near midlength…………………..
  *……………………………………………………………………..Mesephialtites* Rasnitsyn, 1975 [5]
  - Antenna more than15-segmented, 1a-2a present, metasoma widest in rear half, rarely near
  parallel-sided (in males)……..…………………………….*Leptephialtites* Rasnitsyn, 1975 [5]
(1). 1-RS proclivous, short 1r-rs present………………………………………………………
  *………………………………..……………. Brigittepterus* Rasnitsyn, Ansorge & Zessin, 2003 [11]
  - 1-RS reclivous, 1r-rs rarely present…………………………………………………………22
3r-m lost, female metasoma subcylindrical, as long as head and mesosoma combined……..
  *……………………………………………………………….……Symphyogaster* Rasnitsyn, 1975 [5]
  (if correctly figured, *Cephenopsis* might run here but differs in having complete 1r-rs reaching
  base of pterostigma and metasoma ordinary: neither short nor cylindrical)
  - 3r-m lost, female metasoma either longer or not subcylindrical…………………….…….23
1a-2a present……………………………..………………………………………………24
  - 1a-2a lost……………………………………………………………………………….…26
Cells 2-3rm and 1mcu of about same length. 1st metasomal segment big, cylindrical,
  distinctly angled with 2nd in side view…*………………..Micrephialtites* Rasnitsyn, 1975 [5]
  - Cell 3rm usually shorter than 2rm and/or 1mcu. 1st metasomal segment narrowing basal
  …………………………………………………………………………………………….25
Cell 2rm along M much longer than cell 1mcu, and 3rm along M about as long as 1mcu.
  Hind femur very thick…………………………………..…… *Karataus* Rasnitsyn, 1977 [6]
  - Cells 2rm and 3rm not simultaneously as long as above. Hind femur ordinary……………
  …………………………………………………………*Symphytopterus* Rasnitsyn, 1975 [5]
(23). 2r-m oblique, almost reaching 2r-rs. Antenna setaceous (narrowing towards apex).
  Body more elongate*……………………………………..………Karataviola* Rasnitsyn, 1975 [5]
  - 2r-m distant from 2r-rs for about its length. Antenna filiform (not much narrowing apical).
  Body more robust………………………………….….*Trigonalopterus* Rasnitsyn, 1975 [5]

#### Phylogenetic analysis

**Figure 8:**
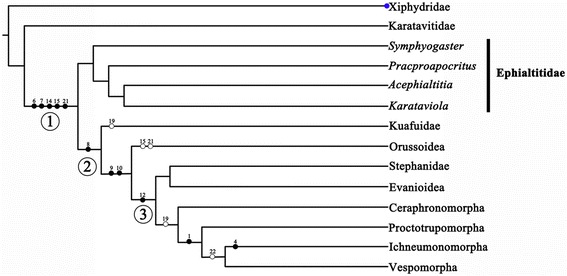
**Results of the phylogenetic analyses as represented by a most parsimonious tree of length 29, CI 0. 82, and RI 0. 83. (●) Nonhomoplasious; (○) homoplasious.**

An analyses using NONA resulted in forty-five most parsimonious trees, each consisting of 29 steps, consistency index = 0.82; retention index = 0.83. (All trees in Additional files 3, 4 and 5: Figures S1, S2 and S3). The most parsimonious tree we used in this paper is shown in Figure 8, with nonhomoplasious and homoplasious characters marked. The major conclusions of our phylogenetic analysis are as follows: Karatavitidae firstly separated from Xiphydridae, as the sister group to the big branch formed by Ephialtitidae, Kuafuidae, Orussoidae, and other groups of suborder Vespina. Four genera *Symphyogaster*, *Pracproapocritus*, *Acephialtitia* and *Karataviola* form a branch Ephialtitidae, which as the sister group of the remaining big clade (Kuafuidae + (Orussoidae + ((Stephanidae + Evanioidae) + (Ceraphronmorpha + (Proctotrupomorpha + (Ichneumonomorpha + Vespomorpha))))). This first big clade supported by the following characters: 1-Rs short, proclined or subvertical, reclined (character 6, state 1), 1r-rs (real or restored when possible) longer than 2r-rs, or lost traceless (character 7, state 1), hind wing m-cu lost (character 14, state 1), hind wing jugal lobe (posterobasal wing area) not delimited (character 15, state 1), first abdominal segment/propodeum fused with metapleuron (character 21, state 1). In the second big clade, Kuafuidae as the sister group to the remain groups is supported by 2r-rs entirely lost (character 8, state 1). In the third big clade, Stephanidae is sister group to Evanioidea, and together they are sister group to the remaining (Ceraphronomorpha + (Proctotrupomorpha + (Ichneumonomorpha + Vespomorpha))), which supported hind wing cell r open or very small (character 12, state 1).

## Discussion

### On the origin and transformation of the propodeal-metasomal articulation

**Figure 9:**
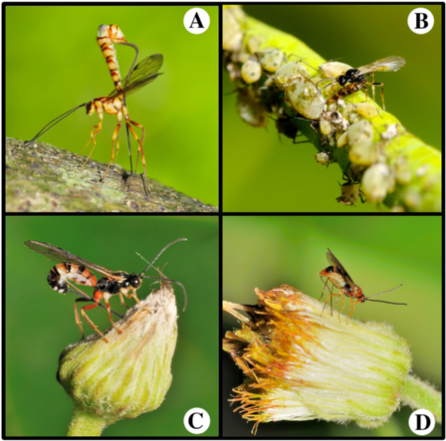
**Typical postures of oviposition of extant wasps (Photos by Jason Shih). A**, The metasoma is raised vertically above the head and mesosoma forming an “L” shape. **B**, The metasoma is bent and parallel to the head and mesosoma forming a “=” shape. **C**, The distal part of metasoma is bent downward from the basal part of metasomal forming an inverted “V” shape. **D**, The metasoma is not bent from the head and mesosoma forming a linear “—” shape.

The first abdominal segment of Apocritan has been incorporated into the thorax as the propodeum. The remaining abdomen, so called metasoma, is connected to this hybrid region via a narrow propodeal-metasomal articulation forming a “wasp waist”, which allows for preferable maneuverability in controlling its ovipositor [27]. By observing some ovipositing extant wasps, representatives shown in Figure 9, we categorize four different postures of oviposition highlighting how various wasps lay eggs by utilizing their propodeal-metasomal articulation and associated capability of controlling their ovipositors. These four typical postures of oviposition are as follows: (1) the metasoma is raised vertically above the head and mesosoma forming a “L” shape (Figure 9A), and the long ovipositor is almost vertically inserted into the branches or flowers from the top of the “L” in order to lay eggs flexibly and accurately into hidden host larvae. (2) The metasoma is bent and parallel to the head and mesosoma forming a “=” shape (Figure 9B), and the ovipositor is used to puncture the host’s gut wall, enter the hemocoel and then lay eggs. (3) The distal part of metasoma is bent downward from the basal part of metasoma forming a inverted “V” shape, the ovipositor and the main body forming an angle from less than 90° to about 90° (Figure 9C), and the ovipositor is inserted into the flowers or plants to lay eggs into hidden host larvae; (4) The metasoma is not bent from the head and mesosoma, forming a linear “—” shape (Figure 9D), and the ovipositor is bent downward into the flowers or plants to lay eggs. Therefore, we believe that the propodeal-metasomal articulation is a very important factor, which influenced the posture of oviposition in Apocrita.

**Figure 10:**
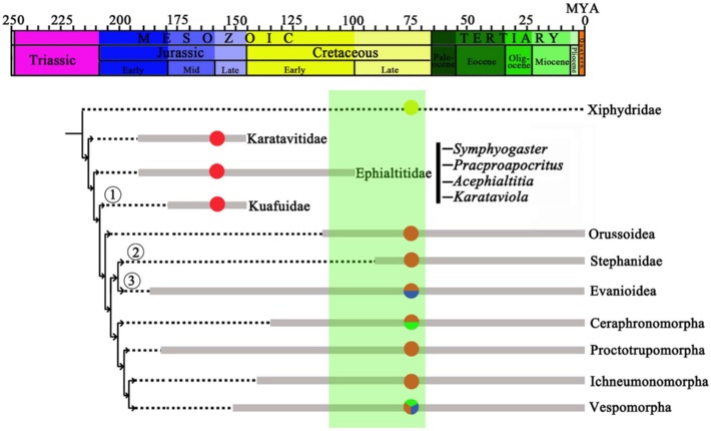
**Cladistic relationships of basal Hymenoptera modified from Figure 8, with fossil data added and the thick lines indicate the known extent of fossils.** Yellow dots represent fungivorous; red dots represent parasitic; red and blue dots represent parasitic and predaceous; red and green dots represent parasitic and phytophagous; red, blue and green dots along branches represent parasitic, predaceous and phytophagous. The light green area represents the period when angiosperms appear in the fossil record and rose to ecological dominance (the Barremian–Cenomanian interval, from about 130–93.6 Ma) (Peralta-Medina & Falcon-Lang, 2012).

After studying the wasp waists of described fossil species of Ephialtitidae, we identified three typical but different propodeal-metasomal articulations: the shape of the first metasomal segment varies from narrow and greatly elongate with distal end broader than the proximal end (*Proapocritus elegans* Rasnitsyn & Zhang, [2]), to slightly broad with the sides straight and subparallel (*Proapocritus densipediculus* Rasnitsyn & Zhang, [2]), then, to transversally broad with the sides nearly straight and subparallel (*Acephialtitia colossa* gen. et sp. nov.). The family Ephialtitidae was interpreted as a stem group of Apocrita demonstrating the origin and transformation of the wasp waist characters of the Apocrita [1,5,28]. Our new results in Figure 8 suggest that Ephialtitidae is the sister group to all other Apocrita (Kuafuidae + (Orussoidae + ((Stephanidae + Evanioidae) + (Ceraphronmorpha + (Proctotrupomorpha + (Ichneumonomorpha + Vespomorpha))))), which add more details to the understanding and made possible to develop a hypothesis of the early evolution of higher Hymenoptera. The hypothesis infers that the broad articulation between the propodeum and metasoma in Ephialtitidae was passed on from a still more primitive family of Karatavitidae (Figure 10) [29,30]. This ground plan structure has gradually transformed into the more and more narrow and mobile articulation mechanism due to course of evolution of the family. Different pathways of this transformation have been observed and interpreted as phylogenetically indicative.

1. A key character of Ephialtitidae, as well as of more primitive taxa (Symphyta, including Karatavitidae), is that the first abdominal segment (propodeum) is more or less convex (bent) transversally but even (little or not at all bent) longitudinally (Figure 11A). The first pathway of transformation of the metasomal attachment is demonstrated by the Jurassic family of Kuafuidae. This time both articulating foramens got narrower accompanied with the propodeum dorsum bent longitudinally (Figure 11B). As a result, the narrow and mobile metasomal articulation appeared low on the propodeum (close to hind coxae), as is typical of the remaining Apocrita, that is, the Aculeata, Ichneumonomorpha, Proctotrupomorpha, and Ceraphronomorpha (including Trigonalidae, Megalyridae and Ceraphronoidea). This pathway is shown as ① in Figures 10 and 11.

**Figure 11:**
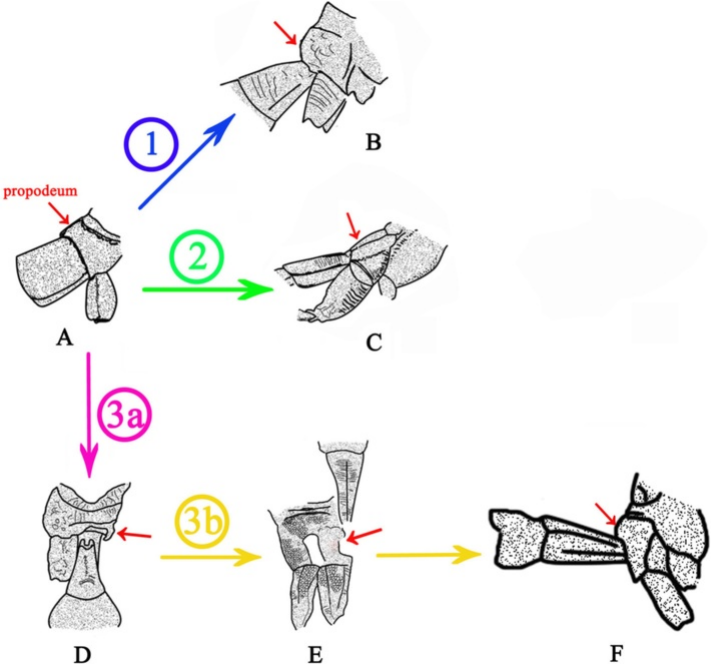
**Transformation of the propodeum-metasomal articulation in basal Apocrita (including Evanioidea)** **. A**, *Acephialtitia colossa* gen. et sp. nov. **B**, *Kuafua polyneura* Rasnitsyn, Zhang, 2010 (Kuafuidae). **C**, *Schlettererius cinctipes* (Cresson, 1880) (Stephanidae). **D**, *Proapocritus sculptus* Rasnitsyn & Zhang, 2010. **E**, *Eosaulacus giganteus* Zhang and Rasnitsyn, 2008 (Preaulacidae). **F**, *Eosaulacus granulates* Zhang and Rasnitsyn, 2008 (Preaulacidae). Red arrows indicate propodeum, encircled numbers denote main transformation pathways, as follows: ① – pathway towards Kuafuidae and further to main taxa of Apocrita; ② – pathway towards Stephanidae; ③ – pathway towards basal Evanioidea.
2. The second pathway of transformation is the pattern (Figure 11A) basically retained in the extant family Stephanidae (Figure 11C), except that the articulatory foramen is often narrow here. The propodeal dorsum persists flat but becomes slanting towards its apex rather than horizontal (a feature not uncommon in Ephialtitidae themselves, cf. Figure 5A). This pathway is shown as ② in Figures 11 and 10.
3. Some other Ephialtitidae (Figure 11D) demonstrate initial steps in a different direction. Their articulatory orifices transformed in a disparate way so as the propodeal one retained wide whilst the metasomal base became narrow and hinged to the hind upper rim of propodeum, leaving membranous most of the hind face of propodeum. Sclerotization of this membranous space from its lower sides towards midline is observable in some Jurassic evanioids of the extinct family Praeaulacidae (Figure 11E). This sclerotization resulted in closure of the propodeum under the metasomal attachment which is the key synapomorphy of Evanioidea (Figure 11F). The third pathway is shown as ③ in Figures 10 and 11.

The above hypothesis suggests three separate pathways of the wasp waist transformation. Concerning our considerations about various ovipositing postures of Apocrita, this poses limitation only on the most sophisticated “=”-posture for both ephialtitid-stephanid and evanioid lineages, as well as on the “L”-posture for only the most basal versions of the ephialtitid mechanism (Figure 11A). The ovipositing posture is evidently dependent on the ovipositor length. For instance, the “=”-posture correlates with the short ovipositor, in contrast to the “L”-posture which is useful only for the long one. This correlation raises a question about the ground plan length of the apocritan ovipositor. The basal apocritan family Ephialtitidae consists of two subfamilies differing, among other characters, in the above character: the long ovipositor was claimed diagnostic of Ephialtitinae, and the short one of Symphytopterinae [28]. Still more basal family Karatavitidae has short ovipositor which might suggest that it is the ground plan character of Ephialtitidae. However, the most plesiomorphic wing venation is known in Ephialtitinae and not in Symphytopterinae [2] which makes possible a reversal nature of the short ovipositor in Symphytopterinae. Yet the recent findings of confirmed Ephialtitinae with the short ovipositor [3] resolve the puzzle and demonstrate that the short ovipositor was probably the ground plan character of both subfamilies of Ephialtitidae.

### On the parasitic larval feeding habit of Apocrita

Based on the information of Table 2, the larval feeding habit of Apocrita is parasitic except for several groups having other feeding habits, such as predaceous (some Evanioidea and Chalcidoidea, many Aculeata), or phytophagous (some Chalcidoidea, many Cynipidae and all bees). We summarize the main hosts of the parasitic wasps, including Coleoptera, Lepidoptera, Homoptera, Hemiptera, Blattodea, Diptera, and Hymenoptera in Table 2. In these host groups, Lepidoptera (butterflies and moths) represent the most diverse and radiated species of plant-feeding insects, while Coleoptera (beetles) is the second most diverse and radiated phytophagous insects [27]. More than 40000 species of Homoptera have been described in the world, all of them are phytophagous, feeding on plant sap. Most species of Heteroptera and Blattodea are phytophagous, only a small part of them are predaceous. Some dipterans (e.g. mosquitoes, midges, dung files, blow flies and hover flies) are pollinators for crop flowers, second only to the bees and wasps [31,32]. The Siricoidea of Hymenoptera always bore into the heartwood to lay their eggs so that their larvae will feed on phloem and xylem of trees [33]. Overall, the feeding habit of most hosts of parasitic wasps is phytophagous. Ephialtitidae were most likely parasitoids, which has been hypothesized based on their long and thin ovipositors that their hosts were xylophagous [28]. In particular, horntail (siricoid) larvae are inferred to be the Vespina (= Orussoidea + Apocrita) ground plan hosts [29]. However, the above inference that the ground plan ephialtitid ovipositor was needle-like thin but short makes the xylophilous hypothesis less reliably supported even though acceptable, for such ovipositor is characteristic now of parasitic wasps with almost any ovipositing habits.

**Table 2:** **Summary of feeding habits of Parasitica**

**Superfamily**	**Feeding habit**	**Main host**
Stephanoidea	Parasitic	Coleoptera, Hymenoptera
Ceraphronoidea	Parasitic	Diptera, Lepidoptera
Megalyroidea	Parasitic	Coleoptera, Hymenoptera
Trigonaloidea	Parasitic	Diptera, Hymenoptera, Lepidoptera
Evanioidea	Parasitic, Predaceous	Blattodea, Coleoptera, Hymenoptera
Ichneumonoidea	Parasitic	Lepidoptera, Coleoptera, Hymenoptera
Plarygastroidea	Parasitic	Diptera, Homoptera, Hemiptera
Cynipoidea	Parasitic, Phytophagous	Diptera, Homoptera, Hemiptera, Hymenoptera
Proctotrupoidea	Parasitic	Diptera, Coleoptera
Diaprioidea	Parasitic	Diptera, Coleoptera
Mymarommatoidea	Parasitic	Homoptera, Coleoptera
Chalcidoidea	Parasitic, Predaceous; Phytophagous	Diptera, Homoptera, Hemiptera, Coleoptera

### On the diversification and extinction of ephialtitid wasps

Based on the information and data summarized in Table 1, ephialtitid wasps existed from the Early Jurassic to the Early Cretaceous, while most of them during the Jurassic. But, they declined drastically from the Jurassic (70 species in 24 genera) to the Early Cretaceous (7 species in 6 genera). It is interesting to note that ephialtitid wasps radiated in the Late Jurassic but, probably became extinct after the Early Cretaceous.

The above considerations suggest addressing to profound changes in vegetation occurred in the Cretaceous while seeking for a possible cause of demise of the Ephialtitidae. It is well known that the non-marine Earth transformed from the gymnosperm- to angiosperm-forested that time. Early angiosperms, vascular flowering plants with seeds enclosed in an ovary, have many primitive features which are considerably different from extant angiosperms [34]. Primitive angiosperms, *Archaefructus liaoningensis*, *Archaefructus sinensis* and *Archaefructus eoflora* from the Early Cretaceous Yixian Formation, are now widely accepted as important fossil angiosperm plants [35-37] Leng and Friis [38] described the other angiosperm, *Sinocarpus decussates,* from the same Formation. These fossil angiosperms provided important information about early angiosperms which co-existed with many gymnosperm and other plants in the same ecosystems [39,40]. During the Early Cretaceous, early angiosperms were usually less popular in the composition of the flora than other plants. Based on literature data, researchers believe that the earliest unequivocal remains of angiosperms are generally thought to be pollen grains in the early Hauterivian (∼130 Ma) [41], and the age when angiosperms appeared in the fossil record and rose to ecological dominance has been considered as the period of the Barremian–Cenomanian interval, from about 130–93.6 Ma [31,32] (Figure 10).

Unfortunately, the hypothesis of ephialtitid extinction caused by changes in the food plants of their insect hosts, even though attractive is not consistent, with the geochronological aspect of the fossil record. Indeed, the vegetation became angiosperm-dominated towards the end of the Early Cretaceous (see above). At the same time, Ephialtitidae appeared as a rare group since the very beginning of Cretaceous and lost the fossil record after the Aptian (Table 1). This pattern correlates rather with changes in the hymenopteran fossil record which demonstrates gradual decrease of a number of other predominantly Jurassic taxa (Praeaulacidae, Megalyriridae: Cleistogastrinae, Mesoserphidae, etc.) and a sudden emergence of many others (various aculeate wasps, Proctotrupidae, Gasteruptiidae: Baissinae, Ichneumonoidea, and some others) [28,42,43]. It is of particular interest that the faunistic transformation might start even before the end of Jurassic (op. cit). These observations imply a different hypothesis, that demise of Ephialtitidae, along with the other taxa listed above, has been driven by competition with numerous new taxa appeared just before or/and soon after the J/K boundary. In particular, the abundant Cretaceous xylophilous Baissinae look like the important competitors of Ephialtitidae, and the same is likely correct for the Cretaceous Ichneumonoidea [18,28,43-45].

## Conclusions

A thorough review of the various types of the propodeal-metasomal articulation of Apocrita suggests that the wide articulation between the propodeum and metasoma in basal Ephialtitidae was passed on from a still more basal family of Karatavitidae and provided three separate pathways of transformation of the wasp waist. In addition, the demise of ephialtitid wasps lagging behind the flourishing of angiosperms suggests that ephialtitid extinction driven by competition with numerous new taxa (e.g. the abundant Cretaceous xylophilous Baissinae and Ichneumonoidea) appeared just before or/and soon after the J/K boundary, rather than the transformed from the gymnosperm to angiosperm-forested led to shortage of food sources for hosts of the larvae of ephialtitids.

### Ethics

The authors declare that the study makes no uses of human, clinical tools and procedures, vertebrate and regulated invertebrate animal subjects and/or tissue, and plants.

## Additional files

Additional file 1: Table S1. Definition of 25 characters and their states. Additional file 2: Table S2. Character state matrix of 25 characters for the 11 taxa included in this study. Additional file 3: Figure S1. An analyses using NONA resulted in 1–15 most parsimonious trees, each consisting of 29 steps, consistency index = 0.82; retention index = 0.83. Additional file 4: Figure S2. An analyses using NONA resulted in 16–30 most parsimonious trees, each consisting of 29 steps, consistency index = 0.82; retention index = 0.83. Additional file 5: Figure S3. An analyses using NONA resulted in 31–45 most parsimonious trees, each consisting of 29 steps, consistency index = 0.82; retention index = 0.83.
