# Supplementary material for: New fossil ephialtitids elucidating the origin and transformation of the propodeal-metasomal articulation in Apocrita (Hymenoptera)
Source: BMC Evol Biol. 2015 Mar 13;15:45. doi: 10.1186/s12862-015-0317-1 (PMC4372304; doi:10.1186/s12862-015-0317-1)
Supplement: Additional file 1: Table S1. — Definition of 25 characters and their states. [file 12862_2015_317_MOESM1_ESM.doc]

**Additional file 1: Table S1 Definition of 25 characters and their states.**

| 1. Medial mesoscutal suture: 0, present; 1, lost.  2. Transverse mesonotal fissure, suture or line: 0, absent; 1, present.  3. Wing fixation apparatus (cenchri + rough area within a loop of 2A vein): 0, present; 1, lost.  4. Costal space at least before Rs base (when C present): 0, wider than C thickness; 1, non-existed or rudimentary.  5. SC: 0, preserved as a crossvein in costal space; 1, traceless lost.  6. 1RS: 0, long, proclined; 1, short, proclined, or subvertical/reclined.  7. 1r-rs (real or restored when possible) in respect to 2r-rs, when identifiable: 0, shorter; 1, longer or lost traceless.  8. 2r-rs: 0, present; 1, entirely lost.  9. 3r-m: 0, present; 1, entirely lost.  10. 2m-cu: 0, present; 1, entirely lost.  11. 2A beyond a_1_-a_2_ crossvein: 0, long; 1, lost entirely or almost so.  12. Hind wing cell r: 0, enclosed, long; 1, open or very small.  13. Hind wing cell 2+3r-m: 0, enclosed; 1, open.  14. Hind wing m-cu: 0, present; 1, lost.  15. Hind wing jugal lobe (posterobasal wing area): 0, delimited by a fold and trucking under the wing at rest; 1, not delimited.  16. Midcoxa with membrane connecting body: 0, lacking clear bay toward medial midcoxal articulation; 1, with that bay.  17. Midcoxa with basal external rim: 0, smooth; 1, indented toward acetabulum of medial midcoxal articulation; 2, forming long diverticulum there.  18. First abdominal segment/propodeum: 0, split longitudinally into halves; 1, entire, sclerotized similarly to thoracic dorsum and not like other abdominal ones, at most with median weak like.  19. First abdominal segment/propodeum: 0, flat dorsally (straight in side view) before metasomal; 1, arching down (convex in side view) toward metasomal attachment.  20. Hind face of the first abdominal segment/propodeum between metasomal and hind coxal bases: 0, widely membraneous; 1, widely sclerotized laterally or entirely.  21. First abdominal segment/propodeum: 0, not fused with metapleuron; 1, fused with metapleuron.  22. Metasomal spiracles 1-6 (abdominal 2-7): 0, large, undoubtedly open; 1, small.  23. Ovipositor: 0, flat saw-like; 1, needle-like thin.  24. Ovipositor: 0, long surpassing abdominal apex at rest; 1, hardly surpassing apex at rest; 2, internalized at rest.  25. Larval diet: 0, vegetarian; 1, carnivorous. |
| --- |
