# Supplementary material for: New fossil ephialtitids elucidating the origin and transformation of the propodeal-metasomal articulation in Apocrita (Hymenoptera)
Source: BMC Evol Biol. 2015 Mar 13;15:45. doi: 10.1186/s12862-015-0317-1 (PMC4372304; doi:10.1186/s12862-015-0317-1)
Supplement: Additional file 2: Table S2. — Character state matrix of 25 characters for the 11 taxa included in this study. [file 12862_2015_317_MOESM2_ESM.doc]

**Additional file 2: Table S2 Character state matrix of 25 characters for the 11 taxa included in this study.**

| Taxa/Character state | 1 | 2 | 3 | 4 | 5 | 6 | 7 | 8 | 9 | 1  0 | 1  1 | 1  2 | 1  3 | 1  4 | 1  5 | 1  6 | 1  7 | 1  8 | 1  9 | 2  0 | 2  1 | 2  2 | 2  3 | 2  4 | 2  5 |
| --- | --- | --- | --- | --- | --- | --- | --- | --- | --- | --- | --- | --- | --- | --- | --- | --- | --- | --- | --- | --- | --- | --- | --- | --- | --- |
| Xiphydridae  Karatavitidae  Orussoidea  *Acephialtitia*  *Praeproapocritus*  *Symphyogaster*  *Karataviola*  Stephanidae  Evanioidea  Kuafuidae  Ceraphronomorpha  Proctotrupomorpha  Ichneumonomorpha  Vespomorpha | 0  0  0  0  0  0  0  0  0  ?  0  1  1  0,1 | 1  1  1  1  1  1  1  1  1  ?  1  0,1  0,1  0,1 | 0  0,1  0  1  1  1  1  1  1  1  1  1  1  1 | 0  0  0  0  0  0  0  0  0  0  0  0,1  1  0 | 0  1  1  1  1  1  1  1  1  1  1  1  1  1 | 0  0  0,1  1  1  1  1  1  1  1  1  1  1  1 | 0  0  -  1  1  1  1  1  1  1  1  1  1  1 | 0  0  1  0  0  0  0  0,1  1  1  1  1  1  1 | 0  0  1  0  0  0  0  1  0,1  0  0,1  1  0,1  0,1 | 0  0  1  0  0  0  0  1  0,1  0  0,1  1  0,1  0,1 | 0  0  0,1  1  0  0,1  0,1  1  0,1  0  1  1  1  1 | 0  0  0  0  0  0  0  1  0,1  0  1  1  1  1 | 0  0,1  1  1  1  1  1  1  1  1  1  1  1  1 | 0  0  1  1  1  1  1  1  1  1  1  1  1  1 | 0  0  0  1  1  1  1  1  1  1  1  1  1  0,1 | 1  ?  1  ?  ?  ?  ?  1  ?  ?  1  -  -  1 | 0  ?  1  ?  ?  ?  ?  2  ?  ?  2  -  -  1,2 | 0  1  1  1  1  1  1  1  1  1  1  1  1  1 | 0  0  0  0  0  0  0  0  0  1  1  1  1  1 | 0  0  0  0  0  0  0  0,1  1  -  -  -  -  - | 0  0  0  1  1  1  1  1  1  1  1  1  1  1 | 0  ?  1  ?  ？  ？  ？  1  ?  ?  1  1  0  0 | 0  0,1  1  1  1  1  1  1  1  1  1  1  1  1 | 1  0,1  0,2  0  1  2  2  0  0  0,1  0,2  0,1,2  0,1  0,1,2 | 0  1  1  1  1  1  1  1  1  1  1  1  0,1  0,1 |

?, state unknown; -, state inapplicable.
