# Supplementary figures and images for: New fossil ephialtitids elucidating the origin and transformation of the propodeal-metasomal articulation in Apocrita (Hymenoptera)
Source: BMC Evol Biol. 2015 Mar 13;15:45. doi: 10.1186/s12862-015-0317-1 (PMC4372304; doi:10.1186/s12862-015-0317-1)

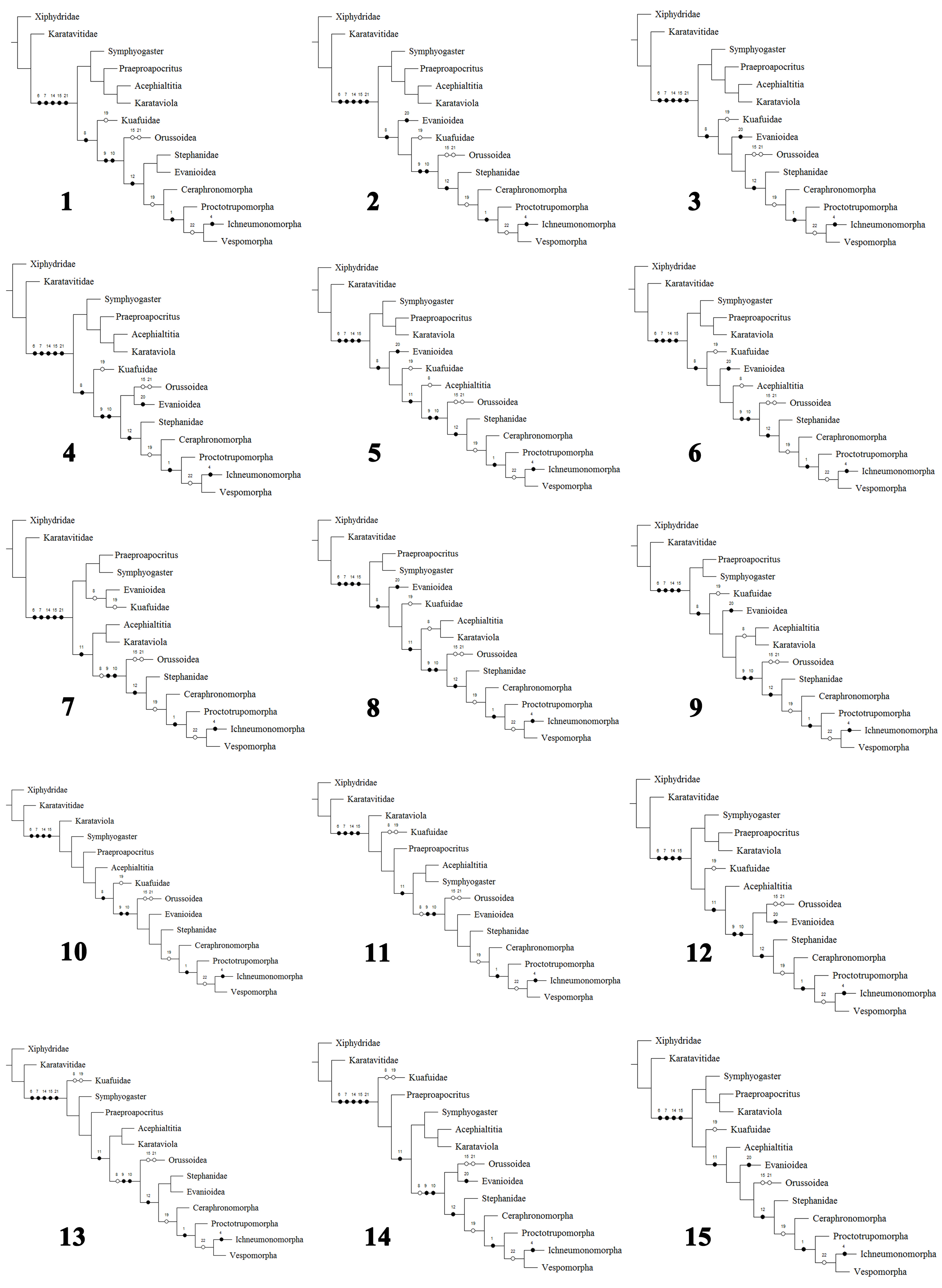

Supplement: Additional file 3: Figure S1. — An analyses using NONA resulted in 1–15 most parsimonious trees, each consisting of 29 steps, consistency index = 0.82; retention index = 0.83. [file 12862_2015_317_MOESM3_ESM.jpeg]

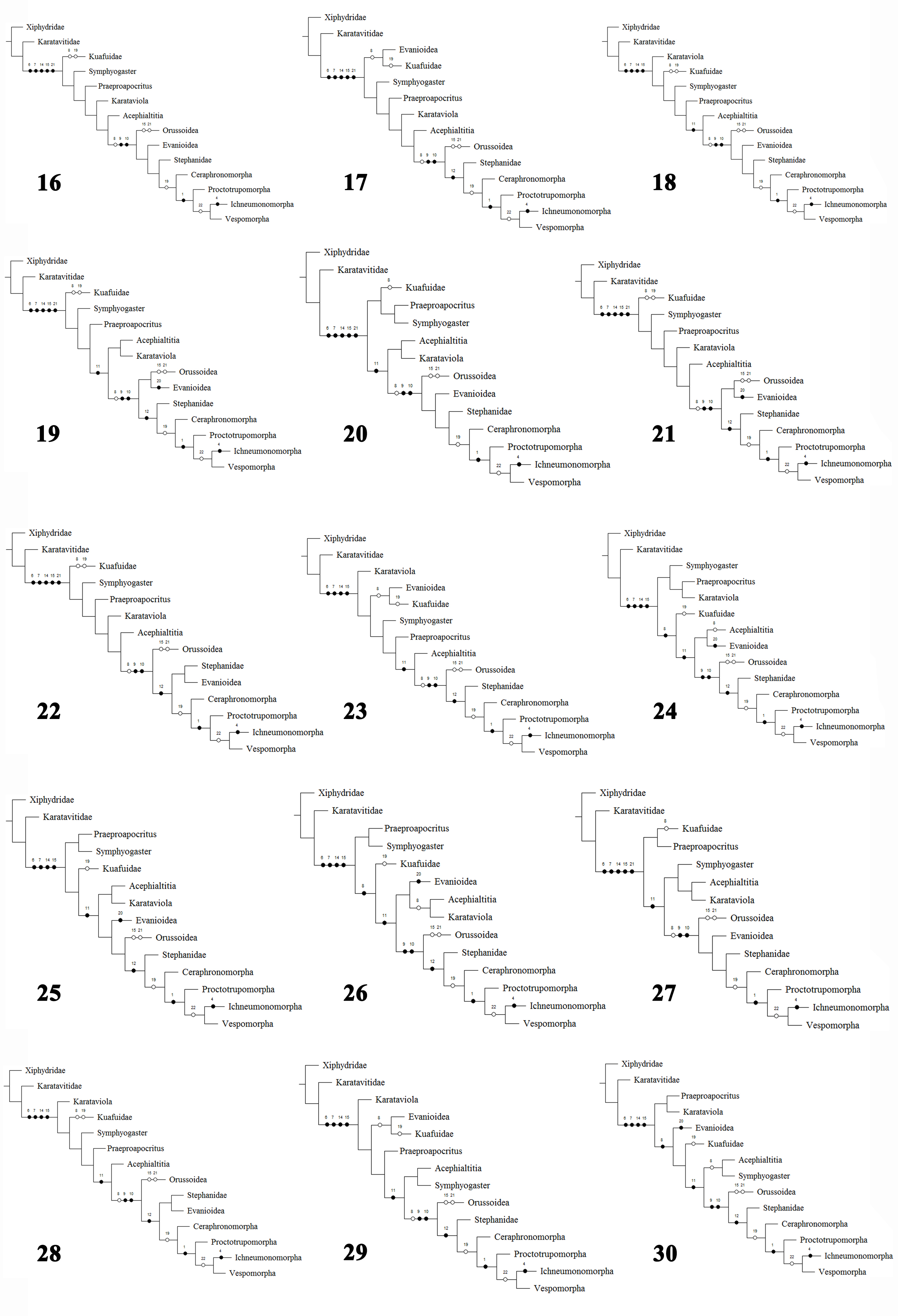

Supplement: Additional file 4: Figure S2. — An analyses using NONA resulted in 16–30 most parsimonious trees, each consisting of 29 steps, consistency index = 0.82; retention index = 0.83. [file 12862_2015_317_MOESM4_ESM.jpeg]

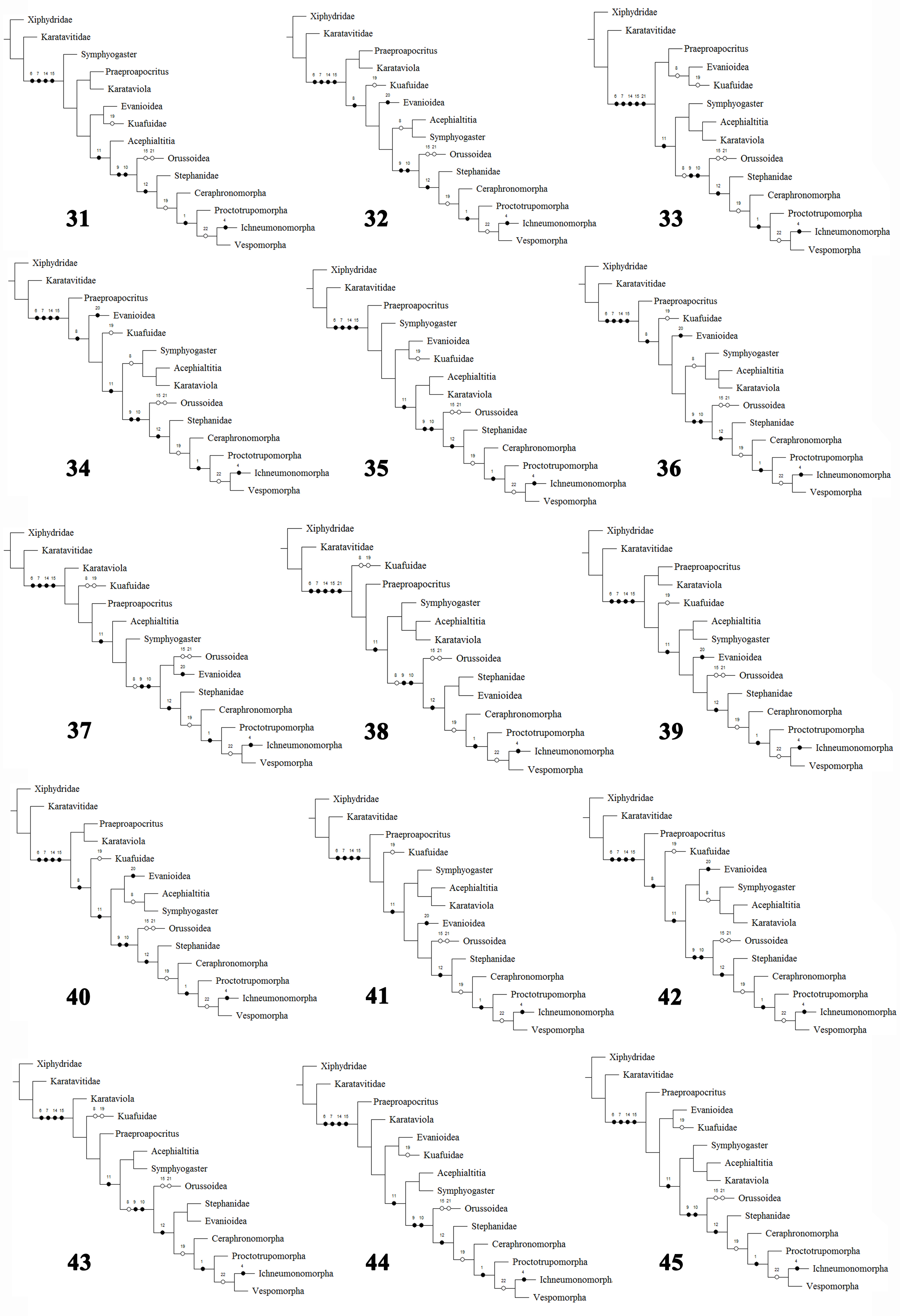

Supplement: Additional file 5: Figure S3. — An analyses using NONA resulted in 31–45 most parsimonious trees, each consisting of 29 steps, consistency index = 0.82; retention index = 0.83. [file 12862_2015_317_MOESM5_ESM.jpeg]
